# A novel glaucomatous representation method based on Radon and wavelet transform

**DOI:** 10.1186/s12859-019-3267-6

**Published:** 2019-12-24

**Authors:** Beiji Zou, Changlong Chen, Rongchang Zhao, Pingbo Ouyang, Chengzhang Zhu, Qilin Chen, Xuanchu Duan

**Affiliations:** 1 School of Computer Science and Engineering, Central South University, Changsha, 410083 China; 2 Hunan Province Engineering Technology Research Center of Computer Vision and Intelligent Medical Treatment, Changsha, 410083 China; 3 The Second Xiangya Hospital of Central South University, Changsha, 410011 China

**Keywords:** Computer-aided diagnosis, Glaucoma detection, Radon transform

## Abstract

**Background:**

Glaucoma is an irreversible eye disease caused by the optic nerve injury. Therefore, it usually changes the structure of the optic nerve head (ONH). Clinically, ONH assessment based on fundus image is one of the most useful way for glaucoma detection. However, the effective representation for ONH assessment is a challenging task because its structural changes result in the complex and mixed visual patterns.

**Method:**

We proposed a novel feature representation based on Radon and Wavelet transform to capture these visual patterns. Firstly, Radon transform (RT) is used to map the fundus image into Radon domain, in which the spatial radial variations of ONH are converted to a discrete signal for the description of image structural features. Secondly, the discrete wavelet transform (DWT) is utilized to capture differences and get quantitative representation. Finally, principal component analysis (PCA) and support vector machine (SVM) are used for dimensionality reduction and glaucoma detection.

**Results:**

The proposed method achieves the state-of-the-art detection performance on RIMONE-r2 dataset with the accuracy and area under the curve (AUC) at 0.861 and 0.906, respectively.

**Conclusion:**

In conclusion, we showed that the proposed method has the capacity as an effective tool for large-scale glaucoma screening, and it can provide a reference for the clinical diagnosis on glaucoma.

## Background

Glaucoma is an irreversible eye disease, by which the vison is permanently damaged. Moreover, glaucoma becomes one of the most common causes of blindness, and more than 80 million people will suffer from this disease by 2020 [1, 2]. Therefore, early glaucoma detection is significant and helpful to save the vision of patients. Clinically, the detection includes intraocular pressure measurement, optic nerve head (ONH) assessment, optical coherence tomography and visual field test [3, 4]. Among these techniques, the ONH assessment is commonly used in the detection. It identifies glaucoma by manually measuring the geometric features of ONH, and is accepted as a significant clinical indicator for glaucoma detection. In fundus images, the ONH is also called optic disc (OD). It is consisted of two distinct regions: a bright region in OD center called optic cup (OC) and the remaining region around OC called neuro-retinal rim. Generally, glaucoma will lead to the structural changes in ONH such as the enlargement of OC, neuro-retinal rim loss, peripapillary atrophy (PPA) etc. ONH assessment can be accomplished by the experts, but manual assessment is influenced by subjective factors and time-consuming. On the contrast, computer-aided methods have the merits of impersonality, rapidity and repeatability [5, 6]. They gain much attention in medical and biological field [7, 8] for association analysis [9–11], feature expression [12, 13] and disease detection [14, 15].

Computer-aided diagnosis methods based on ONH for glaucoma detection were subject to numerous studies in the past. The extensive review published in 2013 [16] cites many works that used template matching, Hough transform, active contours model and level set. We briefly review the most relevant approaches from that study and the methods published later. These methods based on ONH for glaucoma detection can be divided into two categories.

### Glaucoma detection based on geometric parameter measurement

The cup to disc ratio (CDR) is the main risk index used to measure the structure of ONH for glaucoma detection, and neuro-retinal rim loss is also an important evidence in glaucoma detection. Most methods have carried out researches based on these pathological features, including OD localization, OD and OC segmentation, CDR calculation, neuro-retinal rim detection and so on [17–19].

The OD localization can be divided into three categories: (1) OD is the brightest region in the fundus image [20]; (2) OD shows approximate circular or elliptical shape [21]; (3) the blood vessels are used as auxiliary information [22, 23]. The segmentation methods of OD and OC mainly include active contours model [24], morphological method [25], level set [26] and so on. In addition, a series of methods for the measurement of neuro-retinal rim have been proposed, such as ISNT [27] and PPA [28, 29]. On the basis of the above work [30], significant features for glaucoma such as CDR, neuro retinal rim and vascular information around OD were extracted, followed by support vector machine (SVM) and artificial neural network for classification.

These glaucoma detection methods heavily depend on the precise segmentation of OD and OC. However, the structure of OD is liable to be changed by the glaucoma. Moreover, it is difficult to segment the OC region due to its blurring boundary.

### Glaucoma detection based on texture feature

In recent years, glaucoma detection based on texture feature without segmentation is proposed. The texture feature is roughly defined as the spatial variation of the pixel value, which is not specific location on the image [31]. The superiority of earlier mentioned approach is avoiding the probable error in the segmentation, which achieves the goal by learning determining features from the labeled samples and optimizing the machine learning model.

Effectively capturing texture features is the main interest of related work. Bock [32] published research work in this field, which established foundation for the technology. They integrated varieties of texture features for glaucoma detection, including pixel intensity values, Fourier’s coefficients and B-spline coefficients decoding spatial frequency information, and subsequently experimented with SVM to extract glaucoma risk index which indicates the feasibility of the method. Since the texture features can be represented by wavelet transform [33], Dua [31] was concerned about the challenge what wavelet feature was the most discriminative for classification. The three prominent wavelet families that were called Daubechies (db3), Symlets (sym3) and biorthogonal (bio3.3, bio3.5, bio3.7), were adopted for experiments. The features containing pixel values and energy were extracted from wavelet transform, which were subjected to SVM, random forest (RF) and naive Bayes classifier. It proved that db3 and bio3.3 were highly discriminatory. Noronha [34] adopted the perspective of image analysis and recognition, combined the Radon transform (RT) with higher order spectra to represent the fundus images, and employed SVM and Naive Bayes to achieve the purpose of glaucoma classification. Acharya [35] used Gabor filters which possessed the excellent characteristics in the description of the image, and then the mean, variance, entropy and energy features were extracted. After different feature rank and selection strategies, SVM and Naive Bayes completed the ultimate work. Singh [36] thought the area outside of OD in fundus images would introduce interferential information. Therefore, they removed blood vessels from the segmented OD region to improve the classification performance, extracted wavelet features including mean and energy, and used feature reduction and varieties of learning algorithms such as SVM, RF and naive Bayes. In 2017, Zhao [37] combined color distribution, multi-scale Gabor filter and oriented gradient histogram to form multi-channel features representing color fundus images in order to characterize the subtle changes of OD structure and morphology. An RF classifier finally was developed to verify the effectiveness of the algorithm.

In the above literatures, the texture features such as mean and energy are extracted to represent the fundus images, which cannot describe well the complex pattern of glaucoma. Moreover, there is not very convincing that the experimental results are based on the small private dataset. Recently, many deep learning methods are used for medical data analysis, such as convolutional neural networks, recurrent neural network, autoencoder and so on. However, these approaches require large-scale data [38–40]. The aim of this study is to develop a feature representation method to fully and effectively describe on ONH for glaucoma detection. It should provide a reference for the clinical diagnosis on glaucoma.

## Materials and method

### Datasets

Two different datasets were used in our experiments: RIMONE-r2 [41] and Drishti-GS [42]. The RIMONE-r2 consists of 455 ONH images of which 200 are glaucoma and 255 are normal. These images are obtained from full fundus images and have different resolution. This dataset comes from Medical Image Analysis Group and is available online. The Drishti-GS contains 101 fundus images, including 70 glaucoma images and 31 normals. The images are approximately 2047 × 1760 pixels in resolution.

### Method

**Fig. 1:**
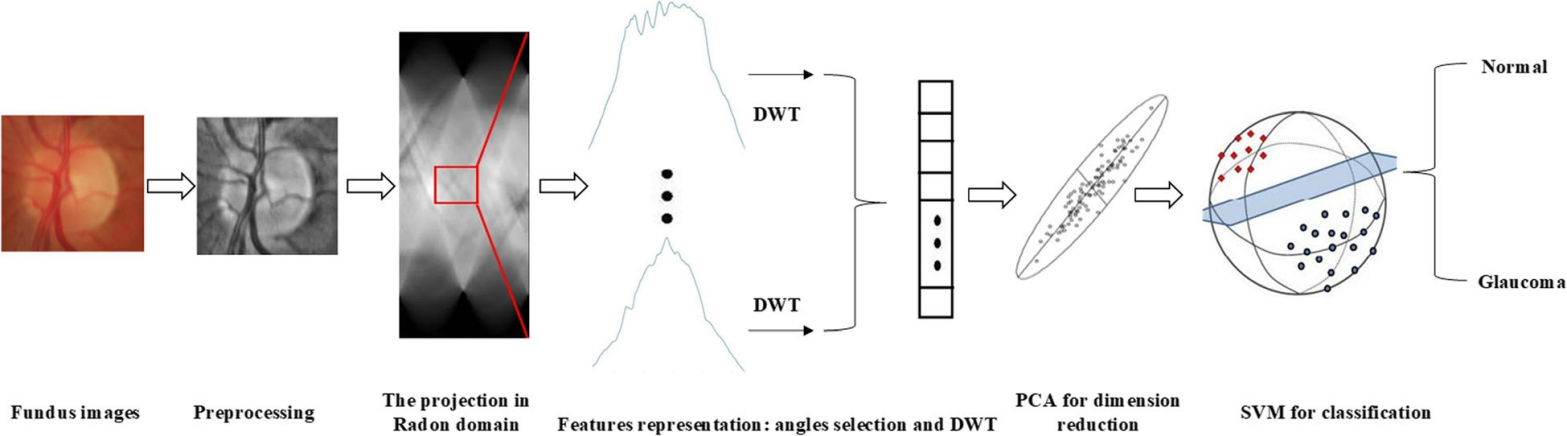
The flowchart of the proposed method

**Fig. 2:**
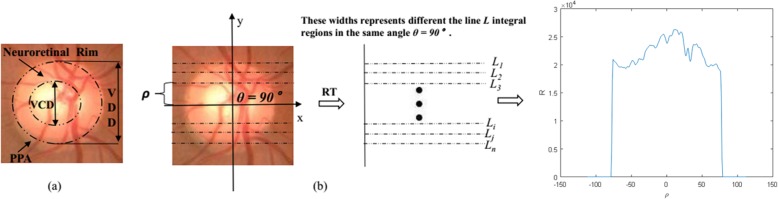
**a** The main measurements on ONH, **b** Radon transform at 90°

In this section, we propose a novel feature representation method for glaucoma detection. Figure 1 gives the flowchart of the proposed method. After the preprocessing, RT and discrete wavelet transform (DWT) is adopted for feature extraction, principal component analysis (PCA) is performed to reduce the dimensions, and SVM is finally developed for automatic detection. In the proposed method, we introduce RT for effective feature representation and avoiding information loss. RT provides a representation of images in the Radon domain, which has the following properties:

1. Constraints Optimization: Generally, CDR is calculated by the ratio of vertical diameter of OC to OD. Neuro-retinal rim loss is not consistent in different directions [13]. Additionally, PPA commonly occurs in the temporal region, as shown in Fig. 2a. Obviously, these characteristics present a trend of radial variation and a 2-dimensional distribution relationship in space. So, it is hard to define an effective descriptor to represent them. By contrast, RT converts the image into 1-dimensional signal and these radial variation and spatial relationship are constrained to a certain extent, as shown in Fig. 2b.
2. Equivalent Features Enhancement: Most of existing methods achieve the comprehensive representation of glaucoma combining CDR, neuro-retinal rim loss and blood vessels features directly. However, RT not only inherently integrates these radial variations, but remains more structural information. Therefore, the features enhancement for glaucoma detection can be done through RT.
3. Dimensionality Reduction: RT is a projection algorithm which can effectively avoid information loss and reduce data dimensions at the same time. For a given image with the size of 300 × 400 pixels, the feature dimension is only about 500 after RT.

## Preprocessing

**Fig. 3:**
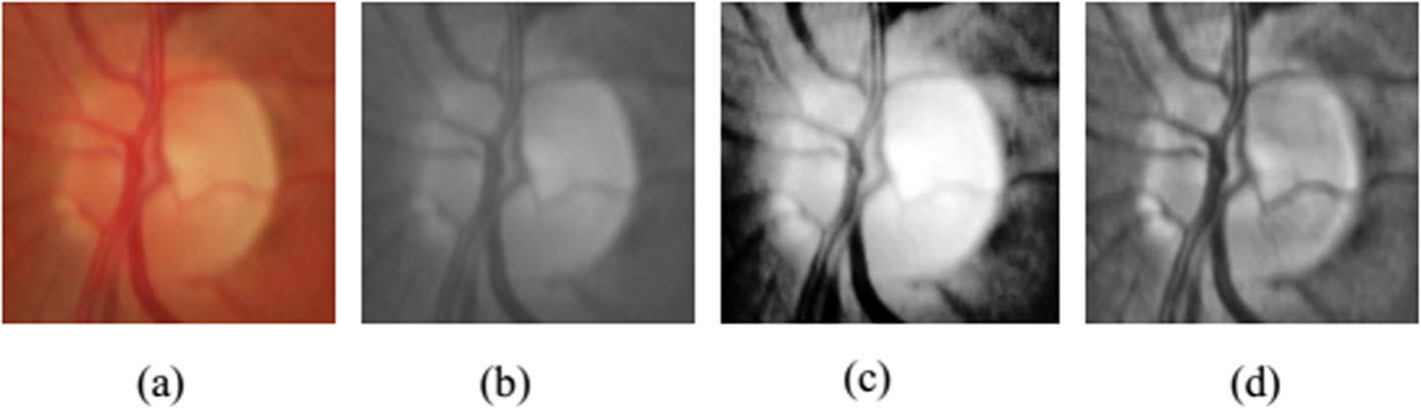
Comparison between common histogram equalization and CLAHE: **a** Color fundus image; **b** Gray-scale image; **c** Common histogram equalization from (**b**); **d** Contrast limited adaptive histogram equalization from (**b**)

The goal of preprocessing is to enhance the contrast and correct the non-uniform illumination of the images. Color fundus images are firstly converted into gray-scale images. Then, the Contrast Limited Adaptive Histogram Equalization (CLAHE) [43] algorithm is followed on these gray images. CLAHE calculates the local histogram of images, and redistributes the intensity of pixels to reinforce image contrast. Figure 3 gives the comparison between common histogram equalization and CLAHE. Figure 3a is the original color fundus image. Figure 3b is the gray-scale image of Fig. 3a. Figure 3c and d are the results of histogram equalization and CLAHE from Fig. 3b, respectively. Compared with the common histogram equalization, CLAHE can enhance the local contrast and retain more information.

### RT for feature projection

In fundus images, the glaucoma is characterized by the significant individual differences and complex symptoms. RT is the projection of the image intensity along a radial line oriented at a specific angle, in which the radial variation of the glaucoma can be well captured. In addition, combining the multi-angles projection may convert the complex visual pattern into discrete signals with high recognition. It also ensures that the transform can represent the original data as much as possible.

For a given image *I*, RT is described as follows [44]:

$$ {\displaystyle \begin{array}{l}R\left(\rho, \theta \right)=\underset{D}{\iint }f\left(x,y\right)\delta \left(\rho -x\cos \theta -y\sin \theta \right) dxdy\;\\ {}\kern1.44em s.t.\kern0.36em \delta (t)=\Big\{\begin{array}{l}1,t=0\\ {}0,t\ne 0\end{array}\end{array}} $$

where *R*(*ρ, θ*) is the result of RT, *f*(*x, y*) is the image intensity at the point (*x, y*), *δ*(▪) is the Kronecker delta function limiting the projection along a straight line, *D* stands for the whole image, *ρ* is the distance of the straight line to the origin and *θ* describes as the angle from the horizontal.

**Fig. 4:**
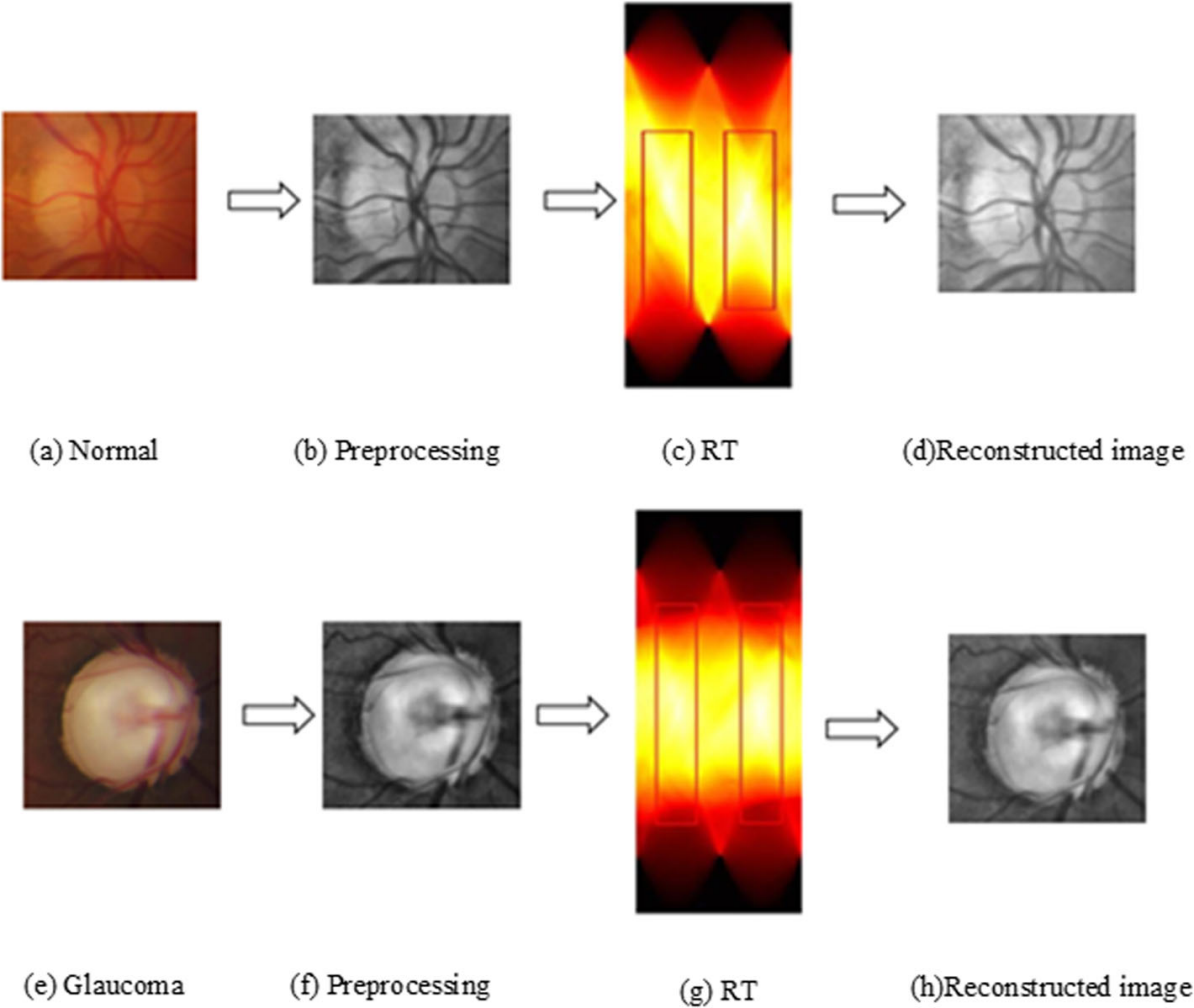
Visualization display in Radon domain and reconstructed image. **a** Normal, **b** Preprocessing on (**a**), **c** RT of (**b**), **d** Reconstructed image of (**c**), **e** Glaucoma, **f** Preprocessing on (**e**), **g** RT of (**f**), **h** Reconstructed image of (**g**)

*R*(*ρ, θ*) varies with the changes of *ρ* and *θ*, which can effectively capture the differences in fundus images. Figure 4 shows the projection results in Radon domain for fundus images. It can conclude that the original information is perfectly preserved by RT from the reconstructed image. It also can be seen that the results reveal high recognition in some area (as shown in red box), which demonstrates that the transform in some specific angles is more distinguishable and superior to characterizing the properties of the original data. Combined with these angles, the complex glaucoma features will be accurately captured, forming an effective descriptor to represent the fundus images. In this research, the combination of nine angles (*θ* = *n ×* 20°, *n* = 1~9) yields the best performances.

### Features representation with DWT

**Fig. 5:**
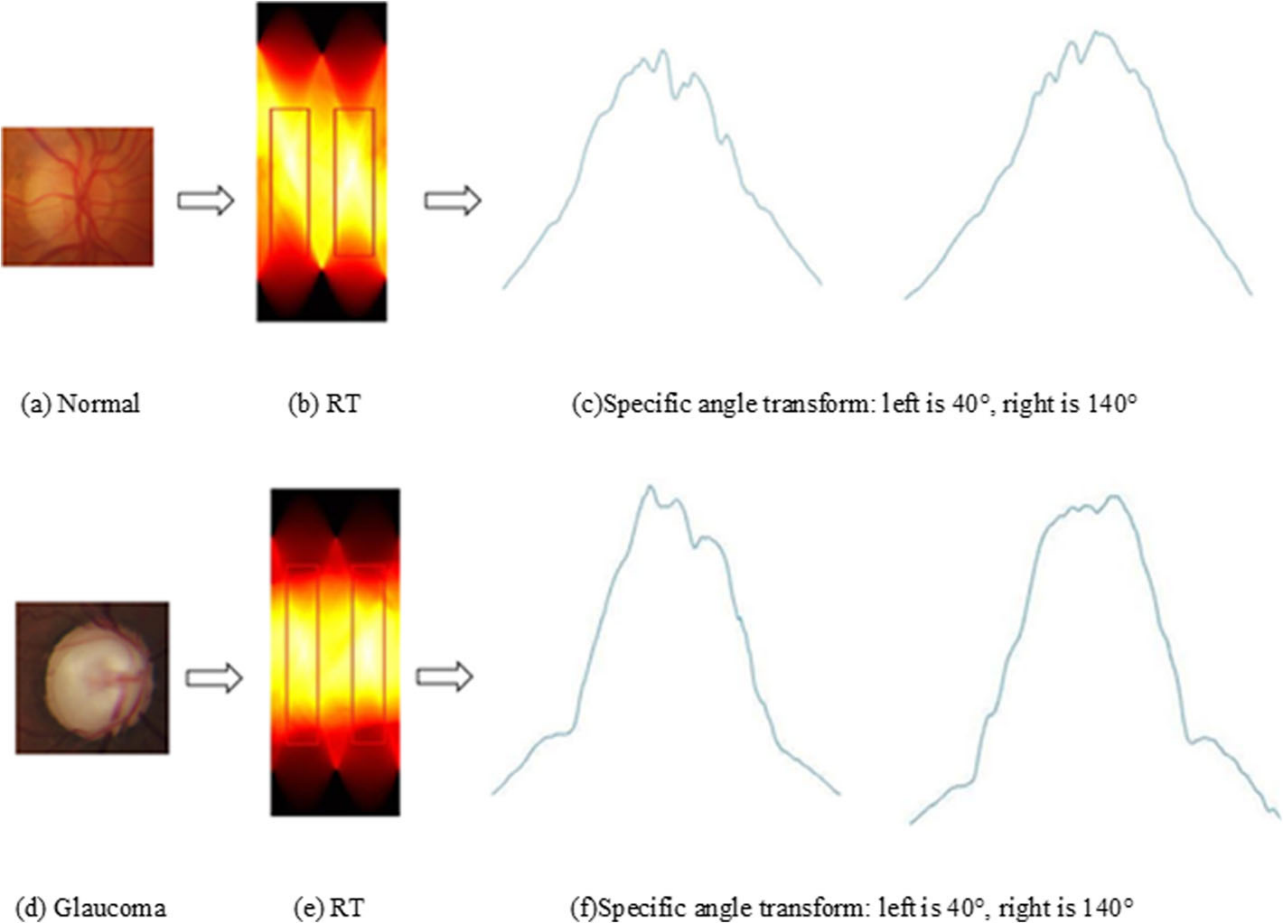
The results of RT at specific angles, in which the differences are concentrated on the middle and jump. **a** Normal, **b** RT of (**a**), **c** Specific angle transform of (**a**): left is 40°, right is 140°, **d** Glaucoma, **e** RT of (**d**), **f** Specific angle transform of (**d**): left is 40°, right is 140°

Because of the individual differences and the complex visual pattern on glaucoma, results of RT varies heavily due to the different angles, which is shown in Fig. 5. Effectively capturing these differences and quantitative representation are the main interest in this section. DWT is an ideal approach for signal processing, and inherits the advantages of the short-time Fourier transform. It has good performance in local analysis of time-frequency domain, and realizes the multi-scale refinement of signal by stretching and translation. Therefore, DWT is employed for feature representation.

**Fig. 6:**
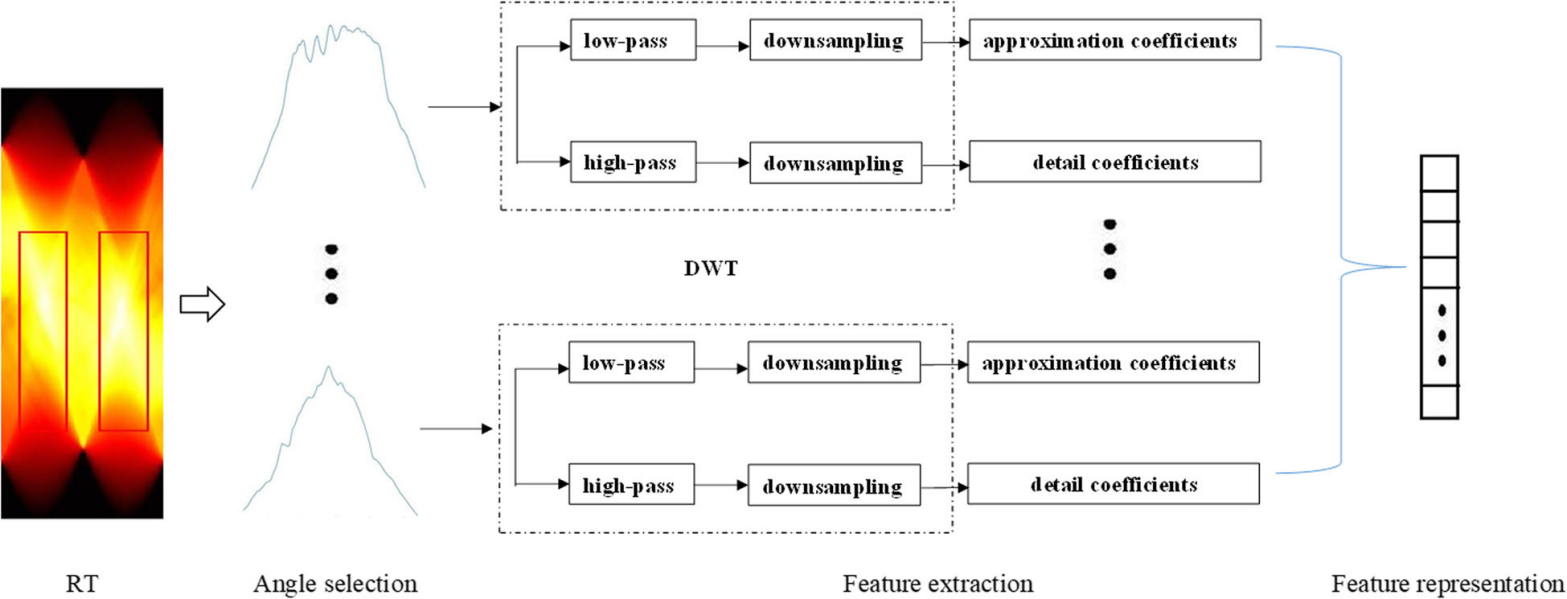
The diagram of feature representation

DWT can convert the results of RT into coefficients and quantify them accurately. For a given discrete signal, DWT is consisted of two sets of coefficients: approximation coefficients and detail coefficients. These coefficients are acquired by convolving the signal with the low-pass filter for approximation and the high-pass filter for detail, and then by down-sampling. Figure 6 shows the process of feature representation. We do not have the coefficients compressed into a single feature such as energy, mean or entropy. On the contrary, the quantitative features are obtained on the analysis of the whole coefficients, aiming at better describing the glaucoma.

### PCA for dimension reduction

The main area suffered from glaucoma is OD and RT is conducted on the whole image, so there is a lot of redundant information. The coefficients acquired from DWT are bound to exist interrelated variables, which has great influence on the classification accuracy. PCA is a statistical method, which converts a group of variables that may be correlated into small number of linear independent variables called principal component by orthogonal transformation. It will find a linear subspace where the original information is retained in the projection data as much as possible. The process of PCA begins with calculating the data covariance matrix, then computes the Eigen values and Eigen vectors of covariance matrix, arranges Eigen vectors in the descending order of Eigen values, and finally projects the original data into the directions of sorted Eigen vectors for reducing dimension and eliminating redundant information [45]. In our research, 94% of the cumulative contributions proportion is chosen.

### Glaucoma detection based on SVM

SVM is developed for detection rather than other classifiers with the following reasons. RT and DWT provide a feature descriptor with low dimensional, and there are not many samples in experimental datasets. In addition, glaucoma lesions are mainly concentrated in OD, so the characteristic differences are relatively gathered in some specific parts of features. SVM is suitable for these complex situations, and is an excellent method in small sample learning. Because of the theory of maximum margin hyper-plane, it can not only grasp the key samples but has good robustness. Moreover, SVM effectively utilizes kernel functions to map the non-linear data into a high dimensional space for classification. Other classifiers like RF are also considered in the research.

SVM is a machine learning algorithm of supervised classification through optimizing the structural risk for better generalization ability. Benefited from the minimization of the empirical risk and confidence interval, it holds the excellent performance for small sample and high dimension learning task. In addition, SVM can dispose of the nonlinear data with kernel function. The kernel function can project the linearly inseparable data into high dimensional space for separating them well. At the same time, the kernel function can transform the computation of inner-product in high dimensional space into kernel function operation, which solves the dimension disaster and lays a theoretical foundation for disposing of the complex classification or regression problem in high dimensional space.

## Results

All experiments were implemented using Matlab R2016a and Libsvm-2.91 [46]. A 10-fold cross-validation approach is utilized for robust statistical evaluation. The performance of our method is evaluated through the accuracy and the area under the curve (AUC) on two public datasets, RIMONE-r2 and Drishti-GS. Sensitivity and specificity are defined as sensitivity = TP / (TP + FN) and specificity = TN / (FP + TN), respectively. TP, TN, FP and FN means the number of true positives, true negatives, false positives and false negatives, respectively.

Firstly, fundus images are transformed into a discrete signal through RT for capturing the complex pattern of glaucoma. Since the database is saved in various resolutions, the bicubic interpolation technique is employed to unified the dimension of signal. Then, the bior1.1 wavelet is used to decompose the discrete signal for feature quantization. After that, all features are subjected to PCA to reduce the dimensionality. Finally, we adopt the SVM and RF classification algorithm to find the best performance. In this research, the best performance is obtained by using SVM classifier with RBF kernel function under ten-fold cross validation.

Table 1 shows the results of the proposed method in glaucoma detection on two different datasets. On the RIMONE–r2 dataset, SVM achieves the highest performance with the accuracy of 86.154% and the AUC of 0.906. But on Drishti-GS dataset, the performance of SVM degrades because of the uneven data distribution. Additionally, RF does not perform well on the conditions of random sampling and inability of data projection, which are not suitable for glaucoma detection.

**Table 1:** Results of the proposed method with 10-fold validation in glaucoma detection

Database	Classifier	Accuracy (%)	AUC
RIMONE-r2	SVM	86.154	0.906
RIMONE-r2	RF	77.100	0.769
Drishti-GS	SVM	74.000	0.732
Drishti-GS	RF	78.000	0.733

Table 2 shows the comparison with other glaucoma detection algorithms. The features called image intensity, fast Fourier transform coefficients and B-spline coefficients are extracted from fundus images. This method reported respectively the accuracy and AUC of 80% and 0.88 [32]. According to its description, we implement this method and evaluate it on RIMONE-r2. We get the accuracy and AUC of 81.319% and 0.89, respectively. Cheng et al. [47] proposed a method for CDR assessment using sparse dissimilarity-constrained coding. They obtained the AUC of 0.83. Maheshwari et al. [48] proposed a methodology for an automated glaucoma diagnosis using fundus images based on empirical wavelet transform. The result showed the accuracy of 81.32% on the RIMONE-r2. In the experiments, we get the results in glaucoma detection with the accuracy of 86.154% and the AUC of 0.906 using RT and DWT. The experiment results show that the proposed method has better performance on glaucoma detection.

**Table 2:** Comparison with other glaucoma detection algorithms

Method	Database	Images	Accuracy (%)	AUC
Bock et al. [32]	Private	575	80.000	0.880
	RIMONE-r2	455	81.319	0.890
Cheng et al. [47]	private	650	–	0.830
Maheshwari et al. [48]	RIMONE-r2	455	81.320	–
ours	RIMONE-r2	455	86.154	0.906

## Discussion

**Fig. 7:**
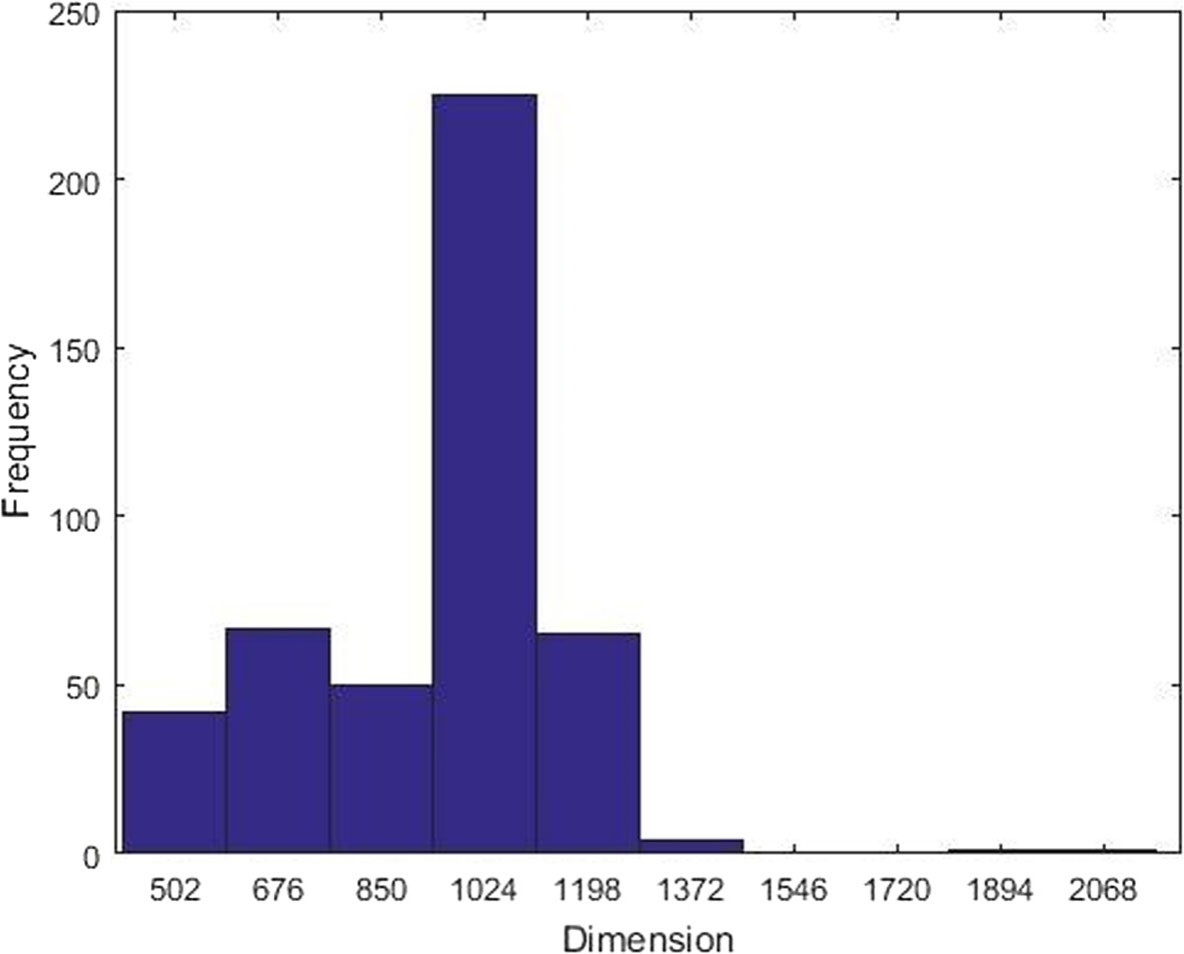
The distribution of frequency in RIMONE-r2

In order to illustrate the performance of the proposed method in Radon domain, we adopt different angles and dimensions to represent color fundus images. Feature dimensions after RT are not the same due to the different image resolutions, as shown in Fig. 7. We choose 6, 9, 18 angles and 600, 800, 1000 dimensions in our experiments, and Table 3 shows the results. Obviously, the most results in accuracy are above 80%, and it means RT can effectively capture and represent the characteristics of glaucoma. Moreover, it arises first and decreases later, which manifests that some of combinations do not contribute to the classification, or even hamper the performance. Among the experiments, the combination of 9 even angles and 690 dimensions is superior to other options in representation for fundus images and obtains the best accuracy.

**Table 3:** Results of different angles and dimensions on RIMONE-r2

Angles	Dimension	Accuracy (%)
6	600	81.198
	800	81.978
	1000	83.736
9 odd	600	81.758
	800	81.539
	1000	79.780
9 even	600	84.396
	800	84.176
	1000	84.176
18	600	82.396
	800	79.341
	1000	83.077
9 even	690	86.154

Additionally, the classification performances with and without DWT are compared, as shown in Table 4. Then, the performances using different biorthogonal wavelets in DWT are compared as well, which is shown in Table 5. From Table 4, the accuracy of the method without DWT is lower than the proposed method. From Table 5, it gets the highest accuracy of 86.154% when the ‘bior1.5’ wavelet is selected. Table 6 gives the comparison results of classification performances with different dimension reduction methods among PCA, Multidimensional Scaling (MDS) [49] and Laplacian Eigenmaps (LE) [50]. Each of dimension reduction methods compresses the extracted features into the same dimension. It is noted that PCA has greater classification performance than the other dimension reduction methods.

**Table 4:** Results with and without DWT on RIMONE-r2

Method	Accuracy (%)
RT	84.396
RT + DWT	86.154

**Table 5:** Results of different biorthogonal wavelets on RIMONE-r2

Wavelets	Accuracy (%)
bior1.1	85.934
bior1.3	85.934
bior1.5	86.154
bior2.2	76.703
bior2.4	76.484
bior2.6	76.703
bior3.3	76.923
bior3.5	76.703
bior3.7	76.923

**Table 6:** Results with different dimension reduction methods on RIMONE-r2

Method	Accuracy (%)
PCA	86.154
MDS	80.879
LE	70.989

Finally, we also compare the results with the different kernel functions of SVM in classification. Common kernel functions are adopted in the experiments such as linear, polynomial and radial basis functions. The results are shown in Table 7. RBF has the best classification performance for the reason of its strong capability in data processing and nonlinear projection. Additionally, SVM classification model appends the penalty coefficient *c* and RBF needs to specify the width parameter of the function *σ*. In this research, the grid search method is used to find the best combination of *c* and *g* in the scope of 2^− 10^~2^10^ with the step of 2^0.5^. The combination (*c* = 11.313 and *σ* = 6.727) is optimal. Moreover, the accuracy increases from 85.274 to 86.154% after utilizing CLAHE, and AUC approximately remains the same. Feature dimensions drop from 6210 to 244 by PCA with 94% of the cumulative contributions proportion, and the accuracy also increases 0.88%. It means PCA removes redundant information and improves the performance of the method.

**Table 7:** Results of different kernel functions in RIMONE-r2

Kernel	Accuracy (%)
Linear	79.780
Polynomial 3	81.319
RBF	86.154

## Conclusion

In this paper, a novel glaucomatous representation method based on Radon and Wavelet transform is proposed. This method extracts the features of fundus images, which can well describe the complex visual patterns. Due to the effective dimension reduction of RT, the proposed method is very quick and efficient and provides strong conditions for the large-scale glaucoma screening. Moreover, AUC of the proposed method is up to 0.906 so that it can provide a reference for the clinical diagnosis on glaucoma.

In the future, we will improve our method on dealing with images in different resolutions, and ensure the method perform well in clinical applications. Additionally, we will study the effect of RT with more exquisite angles, and try to establish a theory for intra-class and inter-class distance in Radon domain, which is aim to promote the performance in glaucoma detection.

## Data Availability

The Drishti-GS dataset is available at the website: http://cvit.iiit.ac.in/projects/mip/drishti-gs/mip-dataset2/Home.php. The RIMONE-r2 dataset is available at the website: http://rimone.isaatc.ull.es.

## Abbreviations

AUC: Area under the curve
CLAHE: Contrast limited adaptive histogram equalization
DWT: Discrete wavelet transform
LE: Laplacian Eigenmaps
MDS: Multidimensional Scaling
OC: Optic cup
OD: Optic disc
ONH: Optic nerve head
PCA: Principal component analysis
PPA: Peripapillary atrophy
RF: Random forest
RT: Radon transform
SVM: Support vector machine
